# Professional experiences in the transition of Cuban-trained South African medical graduates

**DOI:** 10.4102/safp.v63i1.5390

**Published:** 2021-11-22

**Authors:** Munirah Motala, Jacqueline M. van Wyk

**Affiliations:** 1 Department of Clinical and Professional Practice, Nelson R. Mandela School of Medicine, University of KwaZulu-Natal, Durban, South Africa

**Keywords:** foreign medical graduates, FMGs, Cuba, South Africa, challenges, professional experiences, transition theory

## Abstract

**Background:**

Medical educators have been tasked to provide Cuban-trained Foreign Medical Graduates (FMGs) with adequate learning exposures to become integrated into the South African healthcare workforce. International research suggests that FMGs face multiple challenges during the transition from practising medicine in countries other than where they had been trained. The transitional experiences of international FMGs are well documented, but little is known about the challenges faced by Cuban-trained graduates upon reintegration into South Africa. An improved understanding of the challenges will provide insight into how medical educators can best support Cuban trained graduates in their final phase of training in the South African context.

This study explored the challenges experienced during the professional transition of Cuban-trained FMGs with reference to Schlossberg’s transitional theory.

**Methods:**

A qualitative case study was used to interview a purposive sample of 20 Cuban-trained FMGs who studied between January 1997 and December 2007. Data were collected through audio-recorded, semi-structured interviews, which were analysed thematically.

**Results:**

The findings indicate that FMGs’ experienced educational and social stress, which was linked to the transitional situation itself. Challenges during reintegration included bias and discrimination, language, educational differences, and becoming familiar with patients from diverse educational and cultural backgrounds. They drew on peer and institutional support that was mainly informal and varied across disciplines and the medical schools.

**Conclusion:**

Recommendations include a national multidisciplinary consolidated approach to provide personal and professional support at national, institutional, and departmental levels. The creation of mentoring networks will optimise Cuban-trained FMGs’ transitional experiences for returning students.

## Introduction

Foreign medical graduates (FMGs) in South Africa (SA) comprise approximately 1.5% of the South African health sector workforce.^1^ The FMGs who practise medicine in SA are non-South African citizens trained in countries other than SA or South African citizens who trained abroad. One subset of FMGs consists of South African citizens who have completed part of their training in Cuba and then returned to South African institutions for reintegration into the SA’s healthcare context. Studies on the transitional experiences of international FMGs are numerous and highlight the many challenges when practising in countries other than where they had qualified.^2,3,4,5,6,7,8^ There is a lack of published studies on the professional experiences of the Cuban-trained FMGs in SA.

The South African Cuban Medical Collaboration (SACMC) programme started in 1996 as a governmental initiative between the former Cuban president, Fidel Castro and his South African counterpart, president Nelson Mandela.^9,10^ The programme recruits students from high schools in rural and disadvantaged areas of SA to undergo medical training in Cuba. The collaboration was initiated in the post-apartheid era to address the shortage of doctors to serve in rural communities by providing scholarships to deserving students to study medicine in Cuba.^9,10^ The South African students receive pre-medical and Spanish language training during the first year of a 6-year training programme. They then spent the next 5 years studying medicine in Cuban medical training facilities. After that, the students transition to complete the final 18 months of their training at one of the eight South African medical schools.^9,10^ Their undergraduate training programme concludes when they write a Cuban-based theoretical examination in Pretoria, SA. The Cuban-trained graduates are then placed at district-level hospitals in SA to complete a 2-year internship period followed by a year-long community-service period before being eligible for registration as a South African medical practitioner with the Health Professions Council of South Africa.^9^

Undergraduate medical training in Cuba differs greatly from that in SA.^10,11^ Cuban training emphasises health promotion and disease prevention.^10,11^ In contrast, the hospital-based biomedical model is emphasised in SA^10,11.^ Given the nature and scope of the Cuban primary healthcare (PHC) system, concerns have been raised about the suitability of the Cuban training programme to prepare ‘fit for purpose’ graduates to work in the South African healthcare context.^10,11^ For example, general practitioners in the SA PHC context regularly perform clinical skills and procedures, typically only performed by specialists in Cuban settings.^10,11^

Despite the concerns, a 2013-report announced the tenfold expansion of the SACMC programme with the expected return of approximately 1000 Cuban-trained graduates for 5 years from 2020.^12^ Most medical schools are poorly prepared for the influx and the backlog when these students experience academic difficulties. Driven by the need to determine how best educators and medical schools can support the students’ transition, this article explored how Cuban trained graduates adapted to the SA healthcare environment.

Research suggests that medical students encounter multiple challenges whilst transitioning through the various phases of their training.^13,14,15^ Trainee doctors similarly require support to manage the stressful transition from student to doctor.^13^ Furthermore, evidence from qualitative studies suggests that transitions can affect doctors’ performance and ultimately impact the safety of their patients.^13^ To illustrate, a study conducted in Brazil concluded that doctors’ anxiety due to transitioning could result in depression that would influence the way the doctor deals with their profession and their health, and that of their patients.^14^

**FIGURE 1:**
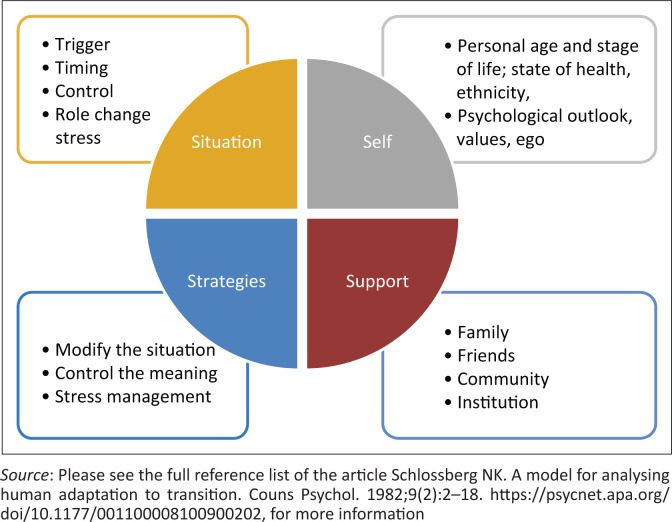
Amended depiction of the four ‘S’. Factors of Schlossberg’s theory.

Transitions in medicine are dynamic processes and refer to how individuals move and adapt from one set of circumstances to another.^15^ A transition refers to an event or non-event that results in a changed relationship or role for the person undergoing the transition.^16,17^ Whilst some people adapt relatively quickly to new situations, relationships and roles, others require more adaptation time to cope with uncertainty and changing roles and relationships.^16,17^ Schlossberg identified four significant sets of factors that may influence a person’s ability to cope with the transitioning process.^16,17^ The four factors, also known as the 4 S’s, require one to consider the *Situation’s impact,* the *Self*, the *Support*, and the *Strategies* during the transition (indicated in Figure 1^16^). Schlossberg’s transition theory provides a possible lens to understand the influence of the transitioning process on this cohort of participants.^16,17^

Schlossberg encourages researchers to evaluate the situational context by looking at factors such as precipitating triggers or factors that initiated the transition, concurrent stressors, and assess how participants view themselves and their responsibilities during the transition and the behaviour which stems from coping with the transition.^16,17^

The second set of factors, the self, relates to personal and psychological characteristics that may affect the individual’s life view, whilst the third relates to social support during the transition. Support includes the roles of family, friends, communities, and institutions in helping to become integrated into the new environment.^16,17^ The final set of factors relates to strategies and responses to modify, cope, control the meaning, and manage the stress that accompanies the transition.^16,17,18^

South African medical schools and educators involved in reintegrating Cuban-trained FMGs during their transition require insight and understanding into the challenges faced by Cuban-trained graduates to coordinate and formalise the support to this cohort of students. Little has been reported on the transitions that Cuban trained students have to make and the challenges, support and strategies that help them reintegrate. We argue that the first step in planning a coordinated response to assist FMGs in SA is to create awareness of their challenges. The next step would be to identify possible collective and institutional interventions to facilitate their successful transition into the SA healthcare context. This study explored how graduates experienced the contextual transition, the challenges they faced during the transition, their support mechanisms, and the strategies that helped them adjust to the new setting.

## Methods

### Study design

An exploratory, descriptive case study was conducted with a purposive sample of 20 South African practising doctors who had enrolled on the SACMC programme between January 1997 and December 2007.^19,20^

### Setting

The study was conducted at the Nelson R. Mandela School of Medicine at the University of KwaZulu-Natal, one of the eight medical schools that accept returning students for reintegration into the SA healthcare system.

### Sampling

Informed by the aim of the study to explore the professional experiences of graduates, a purposive sample of graduates who had undergone Cuban training during the first decade of the SACMC programme was identified as eligible participants. The eligible participants all returned to SA from 2002 to 2012. All the participants (*n* = 20) had enrolled on the SACMC programme from January 1997 to December 2007.^19^

Permission was sought to access a database of students who entered the SACMC programme from a provincial Department of Health (DOH) representative.^19^ The database (*N* = 104) received (November 2016) contained information only on students from KwaZulu-Natal (KZN) who had studied on the programme. The database included the details of the hospitals where the graduates had completed their internship period. Most telephone numbers were no longer in use, but some of the graduates could be contacted through the healthcare facility and permission was sought from them for their participation in the study.^19^

Four of the eligible participants (*N* = 104) initially listed on the database were deceased at the time of data collection. Sixty-one of the possible participants were no longer available on the listed mobile number, and 16 refused to participate. Fourteen participants agreed to the interview. A snowballing strategy was used to request those interviewed to identify other eligible participants of the programme during the specified period.^21^ This strategy yielded additional participants. The purposive sampling strategy allowed for the selection of the participants who had trained on the SACMC programme during the 1997 and 2007 period.^19,20,22^ These same participants had returned to SA between 2002 and 2012, thereby allowing them sufficient period in SA to gain experience in the South African healthcare system.^19^

### Data collection

We used a researcher-administered interview guide for data collection, with each interview taking about 40 min to complete. Most participants (*n* = 19) were interviewed telephonically to accommodate their work schedules, and one was interviewed in a face-to-face session.^19^ The participants were informed of the study and their right to withdraw at any stage. Each participant who agreed, received an information package via email covering the issues to be discussed, and they signed the informed consent. Permission was obtained to audio-record the interviews, which added to the accuracy and richness of the data. Apart from biographical details, the interview explored participants’ professional experiences and challenges upon reintegration into the SA education- and healthcare contexts. They also reflected on the availability of networks and strategies that supported their reintegration. Accuracy was achieved through member checking and the use of field notes. All participants were allowed to express additional opinions and final comments. The transcripts were recorded verbatim and checked for accuracy by the researchers against the audio recordings.

### Data analysis

O’Connor and Gibson’s approach to qualitative data analysis was used for data analysis.^23,24^ The qualitative data were analysed deductively and in relation to the research objectives.^19,23,24^ Each researcher familiarised themselves independently with the data. There were four steps in the process of analysis. The first step included organising the data in response to each research question.^19,23,24^ In the second step, each author independently identified concepts from the transcripts related to each research objective. The concepts were then coded into categories, and finally, the categories identified in step 3 were grouped into overarching themes that answered the research objectives. The authors followed a process of external validation of the categories and themes.^19,23,24^ They also had subsequent meetings to discuss and agree on the process and the reporting of the final themes. Independent analysis by two researchers was used to ensure the credibility of data analysis.^19,23,24^

When conducting the study, the principal author was a clinical coordinator of the fourth year of the Bachelor of Medicine and Bachelor of Surgery (MBChB) programme and lectured students enrolled on the first 3 years of the MBChB programme.

The principal author has been involved with Cuban trained students at UKZN for the past 10 years and has participated in programmes to integrate and orientate the Cuban trained students. None of the participants had any pre-existing personal relationship to the authors, nor was the author an examiner of the student’s clinical examinations or any assessments. Consequently, there were no power-related issues that could have arisen in this study.

The second author is an educational specialist in the school of medicine. The author is involved in quality assurance, faculty development and postgraduate student supervision in the College of Health Science at the university. Hence, the second author has no relationship with the Cuban-trained students at the institution.

## Findings

### Biographical description

A total of six women and 14 men, of average age of 39 years, participated in the study (their ages ranged between 30 and 46 years). At the time of data collection, 13 doctors were married, six were single, and one was divorced. All the participants originated from the rural areas of four provinces, namely KZN, Mpumalanga (MP), North West province (NW), and Limpopo (LP). The doctors had studied medicine at one of the three collaborating Cuban medical training facilities. Nine doctors were based at Cienfuegos, eight studied at Santa Clara, and three at Santa Spiritus in Cuba. After completing the Cuban part of the training, the participants returned to seven of the eight local medical schools and completed their local attachment. Ten participants concluded their training at the University of KwaZulu-Natal (UKZN), two at each of the following four universities: Sefako Makgatho Health Sciences University, Stellenbosch University, University of the Witwatersrand, and the University of Pretoria. One participant completed their training at the University of Cape Town and another at Walter Sisulu University.

The findings are reported in the format of the four factors of Schlossberg’s theory, namely, situation, self, support and strategies.^16,17^ Two descriptive themes were identified in each central theme. The findings describing the *situation* were analysed by identifying triggers and stressors, whilst personal and psychological characteristics were identified in the *self* theme. The two descriptive themes identified in the *support* theme include community and institutional support, whilst *strategies* include evidence of how participants control the meaning of the event and managed the stressors during their transitioning process.^16,17^ The analytical themes, descriptive themes and relevant supportive quotes are shown in Table 1. Descriptive themes were used to classify, describe and compare the experiences reported by the participants, whilst the analytical themes were produced in the theory generating phase when generating possible explanations of the descriptions about the transitioning experience.^25^

**TABLE 1:** The analytical themes, descriptive themes, and supportive quotes.

Analytical themes	Descriptive Themes	Quotes/data from interviews
Situation	Trigger	‘Socially and culturally, there was serious change as I had come from a different environment, coming into SA …’ (Doctor #15, female, 41 years old)
	Situational Stress	‘For so many times, I was never referred to as a SA, only as a Cuban …’ (Doctor #8, male, 35 years old) ‘… Besides the health system, it … being very different and the disease profile being very different. The way their patients are managed is totally different, the society itself was very different … was a big change coming back.’ (Doctor #2, male, 30 years old) ‘In the whole of my training in Cuba, I only saw one patient with HIV, and that patient was a foreigner, he was a tourist …’ (Doctor #11, male, 39 years old) ‘… We had to adjust to the type of patients, that is to say, the level of education- some were educated enough, some were not and especially those who are not, that was my first time interviewing a patient who does not know much about their disease … they don’t know much about the treatment they have been taking for more than 10–15 years, so that was a game-changer …’ (Doctor #4, male, 40 years old)
	Educational stressors	‘… I think the Cuban students when they come back to the SA, to finish their medical career, they need to be realigned with the SA curriculum and to adapt to the SA way of training. Because in Cuba, I don’t think they emphasise certain things. I was confident in some of the areas, but in other fields, I was not comfortable …’ (Doctor #14, male, 41 years old) ‘The transition was not easy because of the Spanish I guess- you had to change your way of thinking, from eating, drinking and sleeping Spanish to eating drinking speaking English, it was tough because you had to change how you think and speak in a space of 3 months to English.’ (Doctor #13, female, 37 years old) ‘My training was in Spanish, and then I come back and speak to a patient in IsiZulu while thinking in Spanish only to present my case in English, it was a nightmare.’ (Doctor #5, male, 36 years old )
Self	Personal	‘You always kind of second guess yourself, you think you are not good enough. Sometimes when you get compared to, you feel like you weren’t trained here, that you weren’t good enough for her …’ (Doctor #13, female, 37 years old) ‘If you meet a doctor who was Cuban-trained in their rooms and they are about to treat you – they will make you feel like a complete person, they go through the basics first – unlike the doctors trained here the mentality they follow, they have the mentality of the specialist. We have a mentality of going back to the PHC thing. I’ve spent a lot of time of doing PHC because it is where we are strongest. We dig deeper looking for the source of the problem rather than looking at current problem we need to go back to the source, solve the problem at the source and the current problem.’ (Doctor #4, male, 40 years old) ‘… And at some time, I felt I was bit [*laughs*] more prepared to deal with patients than [*SA trained doctors*] I think that was based on my training that was patient-centred.’ (Doctor #1, male, 42 years old)
	Psychological	‘… They would label us Cubans and we would have to tell them no we were trained in Cuba, but we are South Africans, but they all saw us as foreigners, so they still had that mindset, that stigma was still there …’ (Doctor #2, male, 30 years old) ‘… We had lots of doctors who were foreign. Doctors from DRC [*Democratic Republic of Congo*], from Argentina. So we were just seen as Cuban doctors, we were one group of many other foreign-trained doctors even though we were South African …’ (Doctor #11, male, 39 years old) ‘… I adapt easily … my background made me tough and I am a tough person.’ (Doctor #18, male, 46 years old) ‘You can only be limited, if you limit yourself.’ (Doctor #3, male, 34 years old)
Support	Community	‘… we had the support system; we really had the support system from our colleagues.’ (SA peers and Cuban-trained peers) (Doctor #15, female, 41 years old) ‘So, for sure one of the coping mechanisms that a Cuban student has to do when they come back- like in my group, what I did- I identify like 1 or 2 people that were my study mates, my buddies, sometimes they will give me their lecture notes for that particular SA disease that I wasn’t sure of. And then I will maybe just use their 4th-year notes just to recap –and as a result, I didn’t have a repeat block.’ (Doctor #1, male, 42 years old)
	Institutional	‘I had one doctor in front [*ahead*] of me who was a Cuban-trained doctor who was able to teach me … We were told which areas we need to focus more on … We had a lot of support from the lecturers as well as the past students from Cuba. I got a lot of support from the SA based students, they supported us so much …’ (Doctor #12, male, 40 years old)
Strategies	Control meaning	‘I am one of those people who adapts easily, I would get close with the people I am with.’ (Doctor #12, male, 40 years old) ‘I realised it has nothing to do with the training as such it has more to do with how you are as a person and how much of how dedicated you are and how much you want to learn.’ (Doctor #4, male, 40 years old) ‘I believe Cuba prepared me well. But there are certain things that are done differently. It was not that I was underprepared; it’s just that I was differently prepared that’s how I found it.’ (Doctor #6, male, 38 years old)
	Actions to prevent/manage stress	‘I think for the first 5 or 6 months, I had to hide the fact that I trained in Cuba because I wanted people to judge me for what I can do, not where I have trained … And it was after six months that people knew I trained in Cuba and people said we can’t believe it because there was a notion that the level of training was substandard as a result, people that are trained in Cuba were not good doctors so to say.’ (Doctor #6, male, 38 years old) ‘I did anticipate that there would be discrimination, and that’s why I hid the fact that I trained in Cuba …’ (Doctor #11, male, 39 years old) ‘As a student, when I came home in vacation, I would split my vacation … example 3 of 6 weeks were spent in the hospital just to have an idea as to what this system is about. That helped me out a lot … It was not compulsory, but it was recommended, and I learned quite a few procedures because I had done 1 or 2 before.’ (Doctor #1, male, 42 years old) ‘I got medical books from SA to acclimatise myself as much as we were doing medicine in Spanish, I would do my part to learn it in English and then get myself prepared so that when the time comes to go to SA, you will not struggle in terms of the terminology and stuff. … Using the internet and stuff, you could see which conditions were much more frequent in SA, medical conditions … and then you focus more on those. to help yourself, you need to grasp both at the same time.’ (Doctor #3, male, 34 years old)

SA, South Africa; HIV, human immunodeficiency virus.

## Discussion

This study explored the professional experiences of Cuban-trained FMGs upon returning to the SA healthcare environment. It sought to understand how graduates experienced the contextual factors during their training, the challenges they faced during the transition period, and the support mechanisms and strategies that helped them adjust to the SA healthcare setting.

The demographic data revealed that most of the participants were black males from rural areas of SA. A total number of six women and 14 men participated in this study. This sample is representative of the racial composition of the general student population in SA when compared with a study published in 2016, which found that most undergraduate medical students in SA universities are black people (38.7%), followed by white people (33.0%), mixed race people (13.4%), and Indian/Asian people (13.6%).^26^

Using Schlossberg’s transition theory, we describe how the *situation, self, support and strategies* frame the transitional and professional experiences of this cohort of doctors.^16,17^

### Situation

In this study, the participants were aware of their expected return date to SA to become integrated into the South African health system and anticipated the social and cultural adjustment as ‘a serious change’. They also anticipated the stressors that were to accompany their transition from a healthcare system that was primarily preventative and patient-centred to one that is disease-focused. The participants in our study were acutely aware of their lack of exposure to patients and diseases such as tuberculosis (TB) and human immunodeficiency virus (HIV), which are prevalent in SA. In this way, our findings are similar to that of an educator-participant who commented on the inappropriateness of Cuban identified core skills for the South African setting.^27^

Educational stressors, including proficiency in English, as the medium of instruction at institutions upon returning to SA, and learning to express themselves with patients from different language and cultural orientations caused some anxiety. Cultural barriers, inadequate knowledge and a lack of cultural norms in caring for patients from diverse cultures was also a concern for international FMGs.^5,8^ Proficiency in English and difficulty translating between Spanish and English when practising in the SA clinical environment were also reported as a challenge for more recent graduates.^27^

Studies suggest that patients’ level of education and the socio-cultural environment may influence the quality of the doctor-patient interaction. A study by McGrath et al. reported FMGs in the Australian healthcare setting being confronted by more educated and informed consumers who demanded higher levels of information and discussion during their consultations.^28^ Participants in the current study thought that their Cuban patients were more informed about their conditions and more assertive when discussing their health than their more challenging South African patients, some of whom were illiterate and displayed relative ignorance about their health.^29^ Whilst international FMGs often report difficulties becoming licenced in the new country, none of these graduates reported the same challenge, which could be explained by the fact that the South African government endorsed the programme.

### Self

In terms of the *Self*, Cuban-trained FMGs developed resilience and the ability to modify their view of the situation. Some participants mentioned that they felt better prepared to interact with patients than their SA counterparts.

At a psychological level, many participants expressed concern about inaccurately being labelled ‘Cuban doctors’ upon their return to the South African setting. In this way, they felt better accommodated in Cuba, where they were viewed as just another student amongst the many cadres of foreign medical students. However, their experiences in the South African environment were in stark contrast, where they faced stigmatisation and discrimination for being Cuban-trained. Our findings concur with that of Sui et al., who also mentioned students’ experiences of anxiety and humiliation upon returning to the South African education system.^27^

The experiences of bias and discrimination are not new for FMGs. The participants in the current study and that of a previous South African study^27^ perceived the bias and discrimination as severe upon re-entering the South African medical school environment. Practitioners in the current study expressed that they expected that the perceived biases and stigmatisation would have subsided after building trust and proving themselves and their clinical abilities after the internship and community service periods.

Graduates from this cohort demonstrated resilience and the ability to modify their view of the *situation*. Having a positive outlook and mindset made it possible to alter their views of the situation and overcome the stigma and labels imposed upon them. The ability to adapt quickly to different circumstances and having the self-belief that one would only be ‘limited if you limit yourself’ was expressed by many participants. This finding of being flexible and adaptive to all medical settings was similar to a previous study that investigated the effectiveness of Cuban training for the South African context.^27^ Despite many challenges, international FMGs also acknowledged personal characteristics, including persistence, flexibility, knowledge and experience gained before working in the host country as factors that contributed to their successful integration in the new country.^4^

### Support

The participants attributed their successful integration to the presence of an informal but good support network amongst themselves. The support includes regular communication between those still in Cuba and those at local medical schools. They shared the challenges and strategies that helped them to succeed during the transition. Having been informed about the lack of clinical exposure, one graduate shared the strategy of volunteering at a local hospital during his vacation to gain greater clinical exposure to diseases that were not prevalent in Cuba. At an institutional level, the participants highlighted the value of an informal support network of South African medical educators and peers, albeit at a departmental or institutional level. They exchanged notes and strategies to help them catch up on topics that were unfamiliar to the FMGs. These notes were also handed down from one generation of Cuban-trained peers to the next. The valuable contribution of this informal network was also highlighted in the study performed by Sui et al.^27^

The collaboration students face stress during their transition resulting in some being able to cope better with the adjustment. Resilience training was documented to help students recover from stressful situations and assist them in enduring ongoing difficulties.^30^ Whilst the current students benefited from mainly informal support, it is also the schools’ responsibility to provide the necessary human and physical resources to help them reintegrate into the local healthcare workforce. Whilst some students demonstrated personal qualities such as optimism and reframing, Johnson mentioned the value of having a nurturing, supportive learning environment for resilience building.^31^ It is argued that trust and a sense of belonging can thrive in a caring environment where authentic relationships can develop and where educators set expectations and facilitate opportunities for students to make meaningful contributions.^31^ As more studies into the challenges have been conducted, it would also be helpful to form a national multidisciplinary consolidated approach to develop a cohesive and comprehensive plan of action to support the returning students as described in the recommendations.

The participants mentioned the value of peer teaching between small teams where local and returning students are teamed and supervised by a local interested supervisor or a practising Cuban-graduate as a mentor. Most of the graduates in the current study volunteered to be part of mentoring dyads or triads and thought they were well-positioned to share life lessons with returning students.

### Strategies

A successful technique used by the participants of this study to overcome challenging situations was to reframe their view of the *situation* by thinking differently about the issue and perceiving it in a different light.^16,17^

This ability helped some minimise the impact of bias and discrimination, as explained by one of the participants who saw himself as ‘differently prepared’ rather than being ‘underprepared’. Some were confident that their patient-centred training had given them an advantage over their South African counterparts when interacting with patients. In this way, our findings support those of Sui et al.,^27^ where Cuban-trained doctors expressed having a different set of core skills and a more ‘hands-on’ approach in managing patients. They also embraced the notion of being adaptable and flexible, and they valued flexibility as being more important to integrate into the South African environment. Participants in the Sui et al. study also emphasised their different core skills,^27^ whilst different clinical exposures for South African practice were not addressed.

The strategies that other FMGs reported as helpful to become reintegrated in other contexts included practical and comprehensive orientation to facilitate the acquisition of local knowledge and the socio-cultural connection within the community; support from faculty mentors and peers; personal and professional support to FMGs along the journey and the use of mentoring facilitated adjustment. Communication and language support helped in the adjustment process during FMG integration. Many FMGs also displayed personal characteristics that supported their adjustment during transitions. These characteristics include persistence, flexibility, knowledge and experiences from interactions with people from different countries.^32^

Medical education and training do not occur in a vacuum, and all doctors should be assisted to work effectively if they are going to work within the same healthcare system. Entering a new system requires that graduates relearn what they thought they knew and learn to apply the knowledge in a more self-directed way when progressing to the clinical platform. The difficulties in transitioning are reported to have lasting effects on the confidence and skills of the qualified practitioner and their interactions with their patients if not addressed appropriately.^15^ In this way, it is detrimental to the new doctor and their patients if they are underprepared for a new transition.^15^

## Limitations of the study

This was an exploratory, descriptive case study of 20 Cuban-trained South African citizens, and the findings may not be transferable to other contexts.^33^ Thus, readers should consider how the findings from this study might unfold in their distinct settings.

Further research on this topic would be helpful as larger cohorts of Cuban-trained graduates are currently being integrated at other institutions, and cross-institutional research will help to understand the extent to which they have had similar transitioning experiences. Because of the lack of literature on the professional and reintegration experiences of Cuban-trained FMGs, there were few publications from which one could draw inferences.

## Conclusion

Graduates from the Nelson Mandela-Fidel Castro Medical Collaboration programme undergo several critical transitions during their intercontinental medical training. They also face extraordinary challenges during the transition periods. These include adapting to a new country; learning Spanish before entering the medical programme; transitioning from the pre-clinical to the clinical phase of medical training; following a traditional curriculum that emphasises holistic and preventative care; interacting with Cuban patients; reverting to English; and being exposed to unfamiliar diseases of South African patients from diverse cultural, demographic and educational levels in the SA healthcare context.

The professional experiences of Cuban-trained FMGs in SA showed similarities with international FMGs, even though these South African citizens had received an 18-month reorientation to the SA healthcare environment.^2,3,4,5,6,7,8,19^ Medical schools in SA have a social obligation to provide South African citizens who received Cuban medical training with appropriate strategies to help them transition into the SA healthcare workforce.

We recommend that a comprehensive plan be devised that firstly will operate at a national multidisciplinary consolidated level to provide appropriate policies to ensure national, institutional and clinical departmental support. This coordinated approach should include all universities training medical students from Cuba, the various departments of health, central government and hospitals associated with the training of students and interns. The support to the Cuban-trained students could then be coordinated centrally and allow for greater efficiency than each university offering orientation and bridging programmes, such as currently being done for returning students.

Secondly, we recommend that mentoring networks be created to provide a structured mentoring programme that includes one of following three options – a peer mentoring option could be provided between students who trained at local institutions and their Cuban counterparts; an individual mentoring track with senior faculty; and a group mentoring option with Cuban-trained doctors who had previously qualified from the institution could also be provided.^34^ A further recommendation would be to incorporate the placement of the Cuban-trained FMGs into ward-based outreach teams. Aligned with the vision for the PHC roll-out, such a PHC team will consist of community healthcare workers to be supported by doctors, nurses and clinical associates for work in community settings.^35^ It is believed that functioning in such a team will provide an enabling clinical mentoring environment for the Cuban trained FMGs to develop their capabilities in a work-based learning setting.^35^

Optimising Cuban-trained FMGs’ transitional experiences should also facilitate the transitional experiences of other foreign-trained doctors who are contributing to an increasingly diverse healthcare workforce in SA.
